# Induced lymphatic sinus hyperplasia in sentinel lymph nodes by VEGF-C as the earliest premetastatic indicator

**DOI:** 10.3892/ijo.2012.1665

**Published:** 2012-10-16

**Authors:** RUEDIGER LIERSCH, SATOSHI HIRAKAWA, WOLFGANG E. BERDEL, ROLF M. MESTERS, MICHAEL DETMAR

**Affiliations:** 1 Cutaneous Biology Research Center, Massachusetts General Hospital and Harvard Medical School, Charlestown, MA 02129, USA;; 2 Department of Medicine A, Hematology and Oncology, University of Muenster, Albert-Schweitzer Campus, D-48129 Muenster, Germany;; 3 Institute of Pharmaceutical Sciences, Swiss Federal Institute of Technology (ETH), 8092 Zurich, Switzerland

**Keywords:** metastasis, sentinel lymph node, VEGF-C, lymphatic sinuses, lymphatic endothelial cells

## Abstract

Research on tumor-induced lymphangiogenesis has predominantly focused on alterations and abnormal growth of peritumoral and intratumoral lymphatic vessels. However, recent evidence indicates that lymphangiogenesis of sentinel lymph nodes might also contribute to cancer progression. In clinical oncology, the sentinel lymph nodes play an important role in diagnosis, staging and management of disease. The prognostic value that may be placed in the analysis of various parameters in tumor-free lymph nodes is still under debate. We, therefore, chose to investigate genetically fluorescent MDA-MB-435/green fluorescent protein human cancer cells transfected to overexpress VEGF-C in a nude mouse model and investigated metastasis, lymph node lymphangiogenesis, lymph node angiogenesis and size of sentinel lymph nodes. The nature of MDA-MB-435, identified as a breast cancer cell line for several decades, has recently been reidentified as being from melanoma origin. Vascular endothelial growth factor-C overexpression induced early metastasis and significantly increased the lymphatic vessel area in sentinel lymph nodes even before the tumor metastasis. At early time-points, expansion of the lymphatic network was observed even though no difference of blood vessel area and lymph node size was detected. These results suggest that primary tumors -via secretion of VEGF-C- can induce hyperplasia of the sentinel lymph node lymphatic vessel network and thereby promote their further spread. In cases of tumor-free lymph nodes the increased lymphatic network of sentinel lymph nodes is a very early premetastatic sign and may provide a new prognostic indicator and target for aggressive diseases.

## Introduction

Tumor-induced lymphangiogenesis has primarily been investigated concentrating on peritumoral and intratumoral lymphatic vessels at primary sites. Studies in animal tumor models have shown that lymphatic vessels promote the metastatic spread of tumors (1–3), and that the induction of lymphangiogenesis could even be used as a prognostic indicator for metastatic risk of human malignant melanoma of the skin (4). The vascular endothelial growth factors (VEGF)-C and -D have been identified as factors predominantly lymphangiogenic via the VEGF receptor-3 (VEGFR-3) (5). Studies have shown that this receptor is expressed on lymphatic endothelial cells of lymphatic vessels (5) and on lymphatic sinuses within lymph nodes (6). Moreover, it has been shown that blocking VEGFR-3 signaling can decrease tumor lymphangiogenesis and cancer spread (7–9).

Previously, were able to show that lymphangiogenesis of sentinel lymph nodes might also play a role in cancer progression. VEGF-A and VEGF-C expressing skin tumors maintained their lymphangiogenic activity after metastasizing to the sentinel lymph node and even induced sentinel lymph node (LN) lymphangiogenesis before the tumor has metastasized (10,11). LN-lymphangiogenesis was also identified in melanoma models after subcutaneous implantation prior to metastasis (12).

In clinical oncology, the sentinel lymph nodes play an important role in diagnosis, staging and management of disease. Especially in breast cancer and melanoma the involvement of regional lymph nodes is an excellent prognostic indicator. But about two thirds of the invasive cancers have no regional lymph node involvement and of those another third will recur (13,14). Studies evaluating the prognostic value of tumor-free sentinel lymph nodes are contradictory (15–17).

Lymph nodes constitute a critical crossroad between drained proteins, antigen-presenting cells, lymphocytes and even tumor cells. Also in the absence of tumor metastasis draining lymph nodes can undergo hyperplasia in number and size (18), because an immune response is also associated with changes of various parameters, such as fluid accumulation, migration and proliferation (19,20).

To investigate the earliest changes of the sentinel lymph nodes we injected genetically modified fluorescent MDA-MB-435/green fluorescent protein human melanoma cancer cells transfected to overexpress VEGF-C or control vector in nude mice. The MDA-MB-435 cell line, which has been reclassified from breast to melanoma, has been derived from the M14 melanoma cell line (21,22). In tumor-free sentinel lymph nodes we determined the lymphangiogenesis, angiogenesis and lymph node size of sentinel lymph nodes. VEGF-C overexpression significantly induced lymphatic sinus hyperplasia in sentinel lymph nodes even before the tumor metastasis. At early time-points, the expansion of the lymphatic network was observed even though no difference of blood vessel area and lymph node size was detected. These results suggest that primary tumors, via secretion of lymphangiogenic factors, can induce hyperplasia of the sentinel lymph node lymphatic network and in case of tumor free non-enlarged lymph nodes might provide a new prognostic indicator.

## Materials and methods

### Cell lines

As tumor cells we used a previously published cell line (1). The MDA-MB-435 cell line, which has been reclassified from breast to melanoma, was derived from the M14 melanoma cell line (21, 22). The two MDA-MB-435 cell lines (American Type Culture Collection, Rockville, MD, USA) were grown in DMEM with 10% fetal bovine serum (FBS) and transfected with the expression construct (pcDNA3.1/EGFP) using the Superfect reagent (Qiagen, Chatsworth, CA, USA). Clone 6 had the highest tumor take and produced lymph node and lung metastasis. Clone 6 was transfected with the human VEGF-C cDNA (23) into a pcDNA3.1/ZEO vector. The transfected cell lines were maintained in media containing 600 *μ*g/ml zeocin and 400 *μ*g/ml geneticin. All animal studies were approved by the Massachusetts General Hospital Subcommittee on Research Animal Care.

### Metastasis assay

Cells were injected bilaterally into the second mammary fat pads of athymic, female, eight-week-old NCR nu/nu mice (2×10^6^ cells/100 *μ*l serum-free culture medium). Two mice of each group (VEGF-C transfected MDA-MB-435 and control vector transfected MDA-MB-435) were sacrificed every two weeks until week ten. The two sentinel lymph nodes and the superficial inguinal lymph nodes were removed from each mouse and paraffin embedded (24). Tumor volume was determined as previously published (25). The smallest and largest tumor diameter were measured every other week, using a digital caliper, and tumor volumes were calculated using the following formula: volume = 4/3 × (1/2 × smaller diameter)2 × 1/2 × larger diameter. Tumor data were statistically analyzed by the two-sided unpaired t-test.

### Immunostainings and immunofluorescence analysis

Sections were stained using antibodies to mouse LYVE-1 (kindly provided by Dr D. Jackson, Oxford, UK; Upstate Biotechnology, Lake Placid, NY, USA), CD31 (BD Biosciences Pharmingen, San Diego, CA, USA), Prox-1 (Covance, Berkeley, CA, USA), F4/80 (Serotech, Raleigh, NC, USA) and corresponding secondary antibodies labeled with Alexa Fluor 488 or 594 (Molecular Probes, Eugene, OR, USA). Cell nuclei were counterstained with Hoechst-bisbenzimide (Sigma-Aldrich). Specimens were examined by using a Nikon E-600 microscope (Nikon, Melville, NY, USA) and images captured with a SPOT digital camera (Diagnostic Instruments, Sterling Heights, MI, USA). At autopsy, all axillary and inguinal lymph nodes were examined for the presence of metastases. In addition, we determined the presence of metastases by fluorescence microscopic analysis and H&E staining for each lymph node.

### Computer-assisted lymph node and lymphatic/medullary sinuses analysis

Representative H&E stained slides of the inguinal and axillary lymph nodes, obtained from the two groups (n=10 for each group) and 4 controls, were analyzed for the highest diameter, sections were examined by a Nikon E-600 microscope (Nikon) and images captured with a SPOT digital camera (Diagnostic Instruments). Further sections were stained with lymphatic and blood vessel markers (LYVE-1/CD31) to examine the lymphatic and blood vessel network. Images were captured with a Spot digital camera (Diagnostic Instruments). Computer-assisted morphometric analyses of the lymph node size, lymphatic network (lymphatic plus medullary sinuses) and blood vessel area were performed using the IP-LAB software (Scanalytics, Billerica, MA, USA). Statistical analyses were performed using the two-sided, unpaired Student’s t-test.

## Results

### Lymphatic endothelial markers of the lymph node sinus endothelium

**Figure 1:**
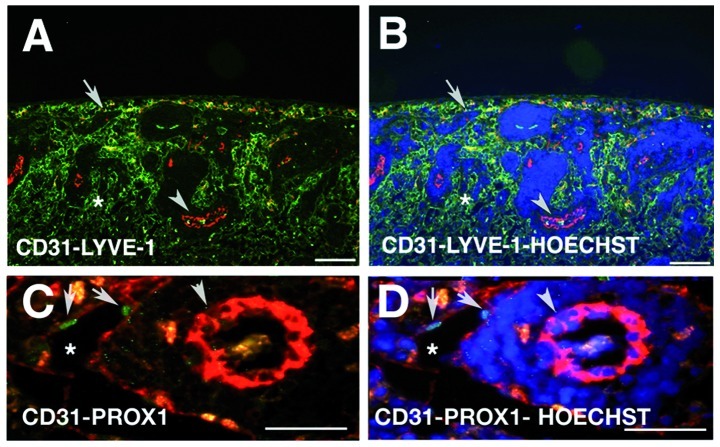
Lymphatic sinuses of lymph nodes express Prox-1. Immunofluorescence staining for LYVE-1, Prox-1 and CD31 depict lymphatic endothelial cells of lymphatic/medullary sinuses and blood vessels in the cortical and paracortical zone of sentinel lymph nodes. (A and B) ^*^Medullary sinuses positive for LYVE-1 (A, green) and high endothelial venules positive for CD31 (A, red, arrowhead). (C and D) ^*^Medullary sinuses positive for Prox-1 (C, green nuclei) and high endothelial venules positive for CD31 (A, red, arrowhead); A and B, scale bar 100 *μ*m; C and D, scale bar 50 *μ*m.

For distinguishing lymphatic endothelium from blood vessel endothelium in lymph nodes for computer-assisted evaluation we identified the lymphatic endothelial cell expression profile. We investigated the expression of known lymphatic markers, using antibodies against Lyve-1, Prox-1 and PECAM-1 (Fig. 1) (reviewed in ref. 26). The lymph node consists of a lymphatic labyrinth filled with lymphocytes (27). Within the lymph node the afferent lymphatic vessel enters the capsule and empties into the subcapsular sinus, which is connected to the trabecular and medullary sinuses (Fig. 1) (28,29). It could be shown that those sinuses are lined with a continued endothelium with long and elaborate intercellular junctions supported by reticular cells (30). Immunofluorescence staining revealed that the lining endothelium of the subcaspsular, lymphatic and medullary sinuses express a lymphatic endothelial profile. Lining endothelium is positive for Lyve-1 (Fig. 1A and B) and Prox-1 (Fig. 1C and D, arrow). Especially, the expression of the most reliable marker for lymphatic endothelial cells, Prox-1, revealed the lymphatic phenotype of the lining endothelium.

Lymphatic sinuses of the cortical and paracortical zone are indicated by the asterisk (Fig. 1A and B). Interestingly, the lymphatic sinuses and medullary sinuses were in close proximity to the high endothelial venules (Fig. 1A and B, arrowhead), but we detected no direct communication.

### VEGF-C does not significantly increase the size of the sentinel lymph nodes

**Figure 2:**
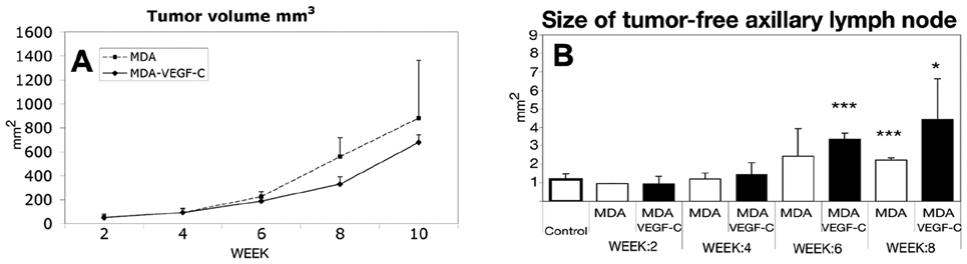
VEGF-C increases tumor-free sentinel lymph node size but not tumor volume. (A) No increase in the tumor growth rate (average size per mouse) of cancer formation was observed in VEGF-C dependency. Only at week 8 there was a significant increase of tumor volume of VEGF-C non-transfected MDA tumors. (B) Increased sentinel axillary lymph node size in mice bearing VEGF-C overexpressing tumors in comparison to control tumors and normal lymph nodes. Bars represent mean values ± SEM.

**Figure 3:**
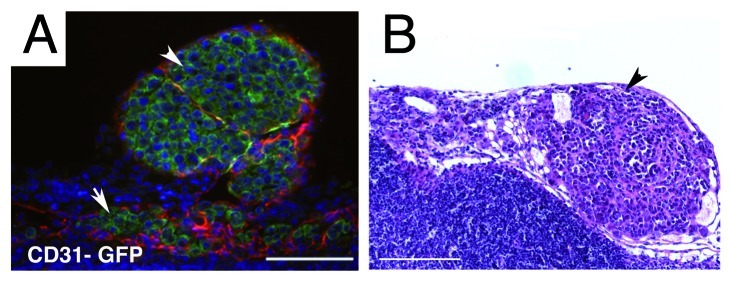
Involvement of draining lymphatics and lymphatic sinuses in sentinel lymph node metastasis. (A) GFP-expressing tumors and immunofluorescence staining for LYVE-1 (green)/CD31 (red) depicts involvement of lymph node lymphatic sinuses and blood vessels. Cell nuclei are stained with Hoechst (blue). Metastatic involvement was evaluated by fluorescence microscopy detecting (A) GFP expressing tumor cells and (B) by H&E, tumor cell dissemination in the subcapsular sinus (A, arrow) and in the afferent lymphatic vessel (A and B, arrowhead); scale bars, (A) 100 *μ*m; (B) 200 *μ*m.

To investigate the effect of VEGF-C on tumor growth and sentinel lymph node size we used the previously published human cell line MDA-MB-435 transfected with human VEGF-C (1). We examined the growth of the tumor volume (Fig. 2A) and found that the average tumor growth rate revealed no significant difference between the VEGF-C transfected and the control cell line (Fig. 2A). To determine if overexpression of VEGF-C leads to differences in sentinel lymph node size, we evaluated the tumor-free sentinel axillary lymph nodes every two weeks (Fig. 2B). The axillary lymph nodes were enlarged at the beginning of week 6, but until week 10 the size of the lymph nodes was not indicative of the presence of metastases. To obtain accurate quantitative analysis of metastases and lymph node involvement, we used cancer cells genetically labeled with GFP, a sensitive method for the direct visualization of micro-metastases. Immunofluorescence for example revealed GFP expressing tumor cells entering the subcapsular sinus (Fig. 3A, arrow) from the tumor containing afferent lymphatic (Fig. 3A, arrowhead). To verify that tumor cells while *in vivo* did not lose their GFP vector every section was in addition evaluated by an H&E stain (Fig. 3B). Metastasis analysis, in agreement with our previously published data, revealed that the incidence of metastases was increased in VEGF-C- overexpressing tumors, as compared with the control tumors. The earliest metastasis in the VEGF-C overexpressing MDA cell line occurred at week 4, while in the control MDA cell line the first lymph node involvement was observed at week 8 (data not shown).

### Lymph node lymphangiogenesis in sentinel lymph nodes

**Figure 4:**
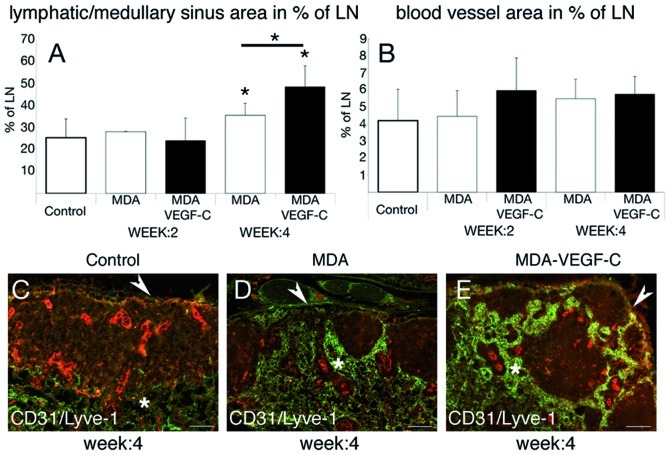
VEGF-C induces lymphatic sinus hyperplasia in sentinel lymph nodes. (A) Morphometric analysis revealed a significant increase of the lymphatic vessel area in sentinel lymph nodes draining VEGF-C overexpressing tumors as compared to the control tumor and normal lymph nodes. Sections were stained for LYVE-1 and CD31. Data are expressed as mean ± SD (^*^p<0.05). (B) Analysis revelaed no increase of the blood vessel area in sentinel lymph node draining VEGF-C overexpressing tumors as compared to the control tumor and normal lymph nodes. Sections were stained for LYVE-1 and CD31. Data are expressed as mean ± SD. (C–E) Immunofluorescence staining for LYVE-1 (green) and CD31 (red) depicts lymphatic vessel area and blood vessels in sentinel lymph nodes. (E) VEGF-C overexpressing tumors induce an increased area of the lymphatic sinuses network in sentinel lymph nodes as compared to the (D) control tumor and (C) normal lymph nodes; subcapsular sinus, arrowhead; ^*^lymphatic sinuses; scale bar, 100 *μ*m.

To investigate the effect of VEGF-C on the draining sentinel lymph node we determined differences of the lymphatic vessel area between the VEGF-C transfected and the control cell line. We evaluated the effect only on lymph nodes without presence of metastatic cells until week 4, an observation point with no visible and statistical difference in general tumor (Fig. 2A) and lymph node size (Fig. 2B). We found that the lymphatic vessel area in percent of the tumor-free lymph node was significantly increased in mice carrying VEGF-C-overexpressing tumors versus control tumors (Fig. 4A, ^*^p<0.05). We further observed no evidence of an increased blood vessel area in percent of the lymph node in mice carrying VEGF-C overexpressing tumors versus control tumors (Fig. 4B). Double-immunofluorescence staining revealed histological changes of the sentinel lymph nodes in mice carrying VEGF-C overexpressing tumors versus control tumors (Fig. 4C–E). In lymph nodes draining VEGF-C transfected tumors the profile of the lymphatic network had a globular appearance with an empty germinal center (Fig. 4C–E). These cup-shaped structures of the lymphatic sinuses (Fig. 4D and E) drained to the medullary sinuses just beneath the deep cortex (Fig. 4C–E). The number of follicles were not statistical different between the two groups (data not shown). In addition, we investigated if tumor associated macrophages revealed any difference in their recruitment, because VEGF-C might also have a direct impact on immune functions (8). VEGFR-3 was detected on macrophages *in vitro* and *in vivo*, and VEGF-C might induce an increased macrophage chemotaxis. To study this we stained the tumors for F4/80, an antigen that is expressed by a majority of mature macrophages. Immunofluorescence analyses revealed no difference in the infiltration of tumor-associated macrophages between the two groups (data not shown).

## Discussion

Lymph nodes are the primary site of immune response and are a critical crossroad, since tumor cells, inflammatory and stroma cells could migrate towards and into them. Although morphological changes of lymph nodes involved and uninvolved in metastasis have been studied in various types of cancers, the prognostic significance of immune response and lymph node size of tumor-free sentinel lymph nodes of breast carcinoma patients is unclear (15–17,31–37). This prompted us to investigate the early morphological changes of lymph vessels in sentinel lymph nodes in the premetastatic situation. In our model using VEGF-C transfected tumor cells versus control cells of the same cell line we observed that the lymph node size and tumor-associated immune response is not predictive for an subsequent metastasis. Both tumors, high- and low metastatic, induced equal lymph node size enlargement. Interestingly, our results suggest that LN-lymphangiogenesis in sentinel lymph nodes still uninvolved in metastasis could be a new early predictor of malignancy. LN-lymphangiogenesis induced by VEGF-C was revealed to be a very early morphological change in sentinel lymph nodes before overt metastasis. Previously, the function and role of VEGF-C was primarily investigated with regard to peritumoral and intratumoral tumor-lymphangiogenesis controlled by VEGFR-3 (5). VEGF-C, as the first lymphangiogenic factor, is proven to be expressed in various cancer cell types (reviewed in ref. 38) and it has been proven to play an active role in the interaction between tumors and lymphatics (1,3). It has been shown that the induction of lymphangiogenesis is a prognostic indicator of the metastatic risk of malignant melanoma of the skin (4), but only a few studies have investigated the effect of tumor derived lymphangiogenic factors on sentinel lymph nodes (6,10–12). We found in a carcinogenesis model that transgenic overexpression of VEGF-A and VEGF-C in the skin induced sentinel lymph node lymphangiogenesis (10,11), also when released by chronically inflamed tissue (39). Our study reported here demonstrates that cancer derived VEGF-C induces sentinel lymph node lymphatic hyperplasia without altering the blood vessel area and lymph node size. Transgenic overexpression of VEGF-C at early time points resulted in an increased lymphatic hyperplasia in VEGF-C draining sentinel lymph nodes in comparison to the control tumors not overexpressing VEGF-C even before the tumor had metastasized.

Based on this observation it could be hypothesized that LN-lymphangiogenesis facilitates tumor cell metastasis, an early event of distant organ metastasis. Hirakawa *et al* recently observed this in VEGF-C overexpressing skin tumors (10), but the role of LN-lymphangiogenesis and its inhibition in the further dissemination of cancer remains largely unexplored. In clinical oncology the size of sentinel lymph nodes has emerged as a predictor next to the extracapsular growth, size of the primary tumor and prescence of lymphovascular invasion, as reported by Van Zee *et al*(40). It has been observed that LN-lymphangiogenesis was associated with an increased frequency of involved non-sentinel lymph nodes in humans (41). The findings suggest that primary tumors, via secretion of lymphangiogenic factors such as VEGF-C induce lymphatic sinus hyperplasia of the sentinel lymph node and thereby might promote their further spread. Recent evidence even indicated that LN-lymphangiogenesis increased lymph flow actively about 20- to 30-fold (6). Although overexpression of VEGF-C induced a more pronounced lymphatic network of sentinel lymph nodes, also the control MDA cell line induced LN-lymphangiogenesis, which indicates that there must be different mechanisms in addition to VEGF-C release. Inflammatory reactions due to tumor necrosis have already been postulated to have an effect on lymph node hyperplasia (42). Especially for inflammatory breast cancers it has been described that they have an angiogenic phenotype and that factors such VEGF-C, VEGF-D and FGF-2 are increased in comparison to non-inflammatory breast carcinomas (43–45). In our xenograft model lymphangiogenic factors produced by macrophages could be a possible explanation, due to the fact that tumor associated macrophages have been identified to produce a broad variety of lymphangiogenic and angiogenic factors (46). We determined the difference of tumor-associated macrophages to exclude that VEGF-C induced a more pronounced secondary response by inducing an increased macrophage chemotaxis, acting via the VEGF-receptor 3 expressed by macrophages (8,46). We found no difference in macrophage infiltration pattern. Studies in which VEGF-A, VEGF-C, FGF-2 or other growth factors in this setting are blocked might answer the question of relative importance of one or more of these factors alone or in concert and could be important for further cancer treatment approaches.

Importantly, our study reveals that the lymphatic network of sentinel lymph nodes should be specifically evaluated by using specific lymphatic endothelial markers. In agreement with the previously published finding by Hattori (47), we recommend that the evaluation of lymph node vessels should not be done by a PECAM-1 (CD31) staining. CD31 is also expressed on the lymphatic sinus network endothelium, but cannot distinguish between the lymphatic and blood vascular system.

Our findings provide additional data to the previously proposed ‘seed and soil hypothesis’ (11), inasmuch as primary tumors might prepare their future metastatic site by producing lymphangiogenic factors that support efficient transport to sentinel lymph nodes, distant lymph nodes and organ sites.

Early changes of the lymphatic sinuses and lymphangiogenesis might predict an unfavorable outcome of an individual carcinoma patient. The lymphatic sinus network of sentinel lymph nodes could even be an important target for future therapies.
